# Numerical Simulation of the Evaporation Behavior of Fe-Mn Heterogeneous Powder in Selective Laser Melting Process

**DOI:** 10.3390/ma17092029

**Published:** 2024-04-26

**Authors:** Xilin Ma, Yaqing Hou, Heping Liu, Hao Qiu, Xiaoqun Li

**Affiliations:** 1 Metallurgical Technology Institute, Central Iron and Steel Research Institute, Beijing 100081, China; 2 Material Digital R&D Center, China Iron and Steel Research Institute Group, Beijing 100081, China; houyaqingwork@163.com (Y.H.); lixiaoqunwork@163.com (X.L.)

**Keywords:** multi-material additive manufacturing, evaporation behavior, mass loss, numerical simulation, selective laser melting

## Abstract

Multi-material additive manufacturing using heterogeneous powders as raw materials is one of the important development directions of metal additive manufacturing technology. The evaporation behavior of heterogeneous powders in the selective laser melting (SLM) process has a significant influence on the accuracy of chemical composition control and the quality of the final product. In this paper, the fusion process of Fe20Mn (80 wt.% Fe and 20 wt.% Mn) heterogeneous powder, Fe and Mn elemental powders, and Fe20Mn pre-alloyed powder is numerically simulated using FLOW-3D^®^ software and partially validated through SLM experimental results. The morphology and the characteristics of the flow field and temperature field in the melt pool for four kinds of powder materials are analyzed. The influence of the elemental evaporation behavior of different powders on the mass loss of the Mn element is discussed. The results show that the excessive accumulation of heat increases the maximum temperature of the melt pool, thus increasing mass loss. The Fe20Mn heterogeneous powder has a wider heat-affected zone and a higher peak value of temperature, nearly 400 K higher than that of the Fe20Mn pre-alloyed powders, which exhibits an intensive evaporation behavior. The mass loss of the Mn element obtained from the SLM experiment for Fe20Mn heterogeneous powders forming parts is more than the Fe20Mn pre-alloyed powders’ forming parts for different laser powers, up to 17 wt.% at P = 120 W. This tendency is consistent with the numerical analysis of the effect of evaporation behavior of Fe–Mn heterogeneous powder during the SLM process. This study provides the necessary theoretical reference and process guidance for realizing the precise control of the SLM composition of a heterogeneous powder in multi-material additive manufacturing caused by evaporation behavior.

## 1. Introduction

Selective laser melting (SLM) is an emerging technology developed in recent years that significantly broadens the application of additive manufacturing techniques [1]. Multi-material additive manufacturing uses a variety of materials to enhance part performance and facilitate unique functionalities. The main processes are selective laser melting (SLM), selective laser sintering (SLS), and electron beam melting (EBM) [2]. In particular, multi-material SLM outperforms its counterparts with superior controllability and a notably reduced heat-affected zone (HAZ) [3], thereby arousing interest in understanding and optimizing the SLM process. Owing to the rapid development of laser scanning, it becomes challenging to observe complex physical phenomena, such as heat transfer and melt pool flow dynamics, which exert a substantial influence on the quality of fabricated components [2,4]. The lack of process control knowledge further confines the broad-scale implementation of this technology.

For metal multi-material additive manufacturing, Fe–Mn damping steel is a potential structural and functional integration material widely used in automotive structural parts and other fields [5]. In the Fe–Mn SLM process, the vaporization of a large amount of the Mn element will lead to the mass loss of Mn and the formation of the defects of the formed parts [6]. The high-energy laser will aggravate the vaporization of Mn. It is difficult to control Mn accurately, and the processing challenge is great [7]. Therefore, it is important to understand the evaporation phenomenon for the Fe–Mn heterogeneous powder of the SLM process. Because the SLM process involves complex physical phenomena, such as heat transfer and melt flow, which are difficult to study fully through experiments, most researchers have used computational fluid dynamics (CFD) modeling to describe the physical behaviors during the SLM process [8,9,10]. For example, Wu et al. [11] studied the influence of evaporation on the flow behavior and the volume of the melt pool and demonstrated the necessity of considering evaporation. Shrestha and Chou [12,13] developed a three-dimensional mesoscopic model to study the effect of powder properties on molten pool dynamics. However, the evaporation effects were usually ignored. Further research by Khairallah and Anderson [14,15] considered more physical phenomena, including Marangoni convection and evaporation recoil pressure in the SLM process. The results showed that the evaporation recoil pressure and Marangoni convection had a significant effect on the flow of the melt pool, and a deep and narrow depression collapse made it easy to form the pores’ defects. Dai [16] studied the melt pool dynamics of TiC/AlSi10Mg SLM. The powder bed was regarded as continuous rather than discretely initializing the powder particles. However, there is a lack of information regarding the characteristics of the melt pool and the impacts of the evaporation behavior on the mass loss of different elements of Fe–Mn heterogeneous powders in the SLM process.

In this study, a mathematics model based on computational fluid dynamics software FLOW-3D v12.0^®^ is developed to simulate the characteristics of the flow field and temperature field of Fe20Mn heterogeneous powder, Fe20Mn pre-alloyed powder, and Fe and Mn elemental powders during the SLM process. The experimental tests for Fe20Mn heterogeneous powder and Fe20Mn pre-alloyed powder are performed to verify this model. The predicted flow field and temperature field for four kinds of powder materials are compared, and the influence of the elemental evaporation behavior of different powders on mass loss during the SLM process is discussed. It is expected that the present study can provide a theoretical basis for laser processing methods and quality accuracy control for heterogeneous powder additive manufacturing.

## 2. Numerical Analysis Method

### 2.1. Mathematical Model

#### 2.1.1. Powder Bed Initialization Model

The Discrete Element Method (DEM) is employed to generate a distribution of particles and to initialize the powder deposition for the first scanning layer [17]. The powder information is then passed into a CFD model to study the interactions between the laser beam and the powder, including calculations of multi-phase flow, surface tension, melting and solidification, gravity force, recoil pressure, and adaptive Gaussian heat sources. The movement of a particle in the DEM model can be described as follows [18,19]:

(1) $$m_{i}\frac{dv_{i}}{dt}=\sum F_{c,i}+m_{i}g$$

(2) $$\frac{d\left(I_{i},\omega_{i}\right)}{dt}=R_{i}\cdot {\sum M}_{c,i}$$

where $m_{i},I_{i},v_{i}$, and $\omega_{i}$ are the mass, moment of inertia, translational velocity, and angular velocity, respectively; $F_{c,i}$ is the contact force; $M_{c,i}$ is the contact torque; $R_{i}$ is the rotation matric from the global to the local coordinate system; *t* is the particles falling time; and g is the gravitational acceleration.

#### 2.1.2. Melt Pool Model

**Figure 1:**
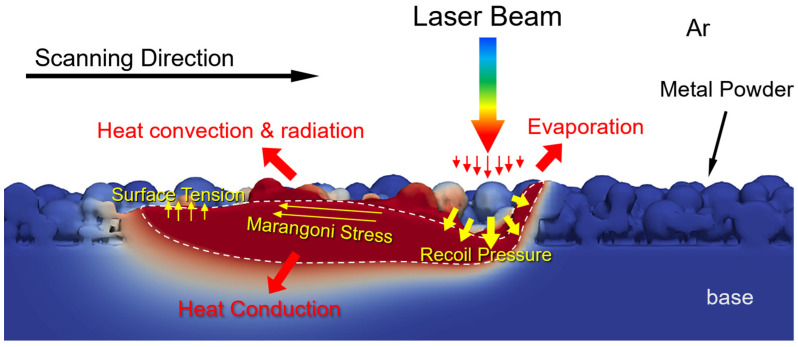
Schematic representation of evaporation dynamics during the laser scanning process.

Figure 1 shows a schematic representation of evaporation dynamics during the laser scanning process. There are complex physical phenomena, such as heat transfer and melt flow in the SLM process, and the CFD model is used to describe the complicated physical behaviors. To simulate the fluid flow in the melt region and determine the temperature distribution, it is necessary to solve the conservation equations. The molten metal is assumed to be a Newtonian incompressible flow. The simulation domain contains two immiscible phases: molten metal and surrounding air. The VOF method is used to track the phase interface, and for a two-phase flow, the mass balance equation is expressed as [20]

(3) $$\frac{\partial }{\partial t}\left(\alpha_{F}\rho_{F}\right)+\nabla \cdot \left(\alpha_{F}\rho_{F}u\right)=-{\dot{m}}_{lv}$$

where $\rho_{F}$ is the fluid phase density, $\alpha_{F}$ is the volume fraction of the fluid phase, ${\dot{m}}_{lv}$ is the volume rate of evaporation mass loss, and u is the fluid phase velocity.

The momentum conservation equation of the SLM melt pool can be expressed as [17]

(4) $$\frac{\partial \rho u}{\partial t}+\nabla \cdot \left(\rho uu\right)=-\nabla p_{e}+\nabla \cdot \overset{̿}{T}+F_{b}+F_{a}{+F}_{m}+F_{t}+F_{P}$$

(5) $$F_{b}=\bar{\rho }g\beta_{L}\left(T-T_{m}\right)$$

where $p_{e}$ represents the pressure. $\overset{̿}{T}$ represents the viscous stress tensor. $F_{b}$ is the buoyancy term, g is the acceleration of gravity, $\beta_{L}$ is the volume expansion coefficient, and $T_{m}$ is the melting temperature. $F_{t}$ is the momentum source phase of the solidification process.

(6) $${F_{t}=-K}_{C}\left(\frac{{\left(1-f_{L}\right)}^{2}}{f_{L}^{3}+C_{K}}\right)u$$

where $K_{C}$ is the mushy region constant, $f_{L}$ is the liquid fraction, and $C_{K}$ is a small constant to avoid a division by zero.

In the momentum equation, the surface tension $F_{a}$, Marangoni force $F_{m}$, and recoil pressure $F_{P}$ are all surface forces at the free interface.

(7) $$F_{a}=\sigma \kappa n$$

(8) $$F_{m}=\frac{d\sigma }{dT}\left(\nabla T-n\left(n\cdot \nabla T\right)\right)$$

where σ, κ, and n are the surface tension coefficient, surface curvature, and surface normal vector, respectively, and dand σ/dT is the surface tension coefficient.

The energy equation of the SLM process simulation can be described by

(9) $$\frac{\partial \rho H}{\partial t}+\nabla \cdot \left(\rho uH\right)=\nabla \cdot \left(k\nabla T\right)+S_{H}+S_{e}$$

where ρ is the material density, t is the time, H is the enthalpy, k is the thermal conductivity, $S_{H}$ is a self-adaptive volumetric heat source, and $S_{e}$ represents the evaporation heat dissipation.

The evaporation heat dissipation is mainly caused by the evaporation of a certain amount of heat, which is proportional to the evaporation flux. The evaporation flux $J_{m}$ can be expressed by the following [21]:

(10) $$J_{m}=F_{P}\sqrt{\frac{M}{2\pi RT}}$$

where $F_{P}$ is the evaporation recoil pressure, M is the relative atomic mass, and *R* is the gas constant.

To accurately simulate the evaporation phenomenon in the laser melting process, the model of evaporation pressure must be taken into account. This phenomenon is simulated by applying pressure to the interface during evaporation, which is solved by Equation (11):

(11) $$F_{P}=P_{0}exp\left\{\left.B\left(1-\frac{T_{v}}{T}\right)\right\}\right.$$

$T_{v}$ is the saturated temperature, *T* is the temperature, $P_{0}$ is the saturated vapor pressure, and *B* is the coefficient.

### 2.2. Geometric Model and Calculation Conditions

**Figure 2:**
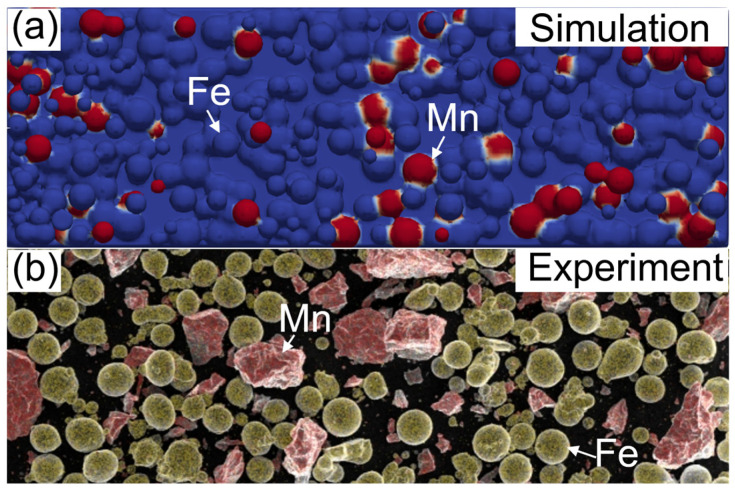
Fe20Mn heterogeneous powder particle distribution: (**a**) simulation results; (**b**) experimental results.

**Figure 3:**
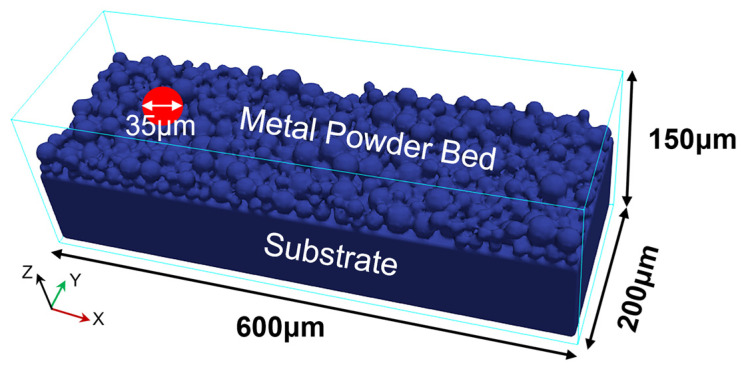
Schematic diagram of the computational domain.

**Figure 4:**
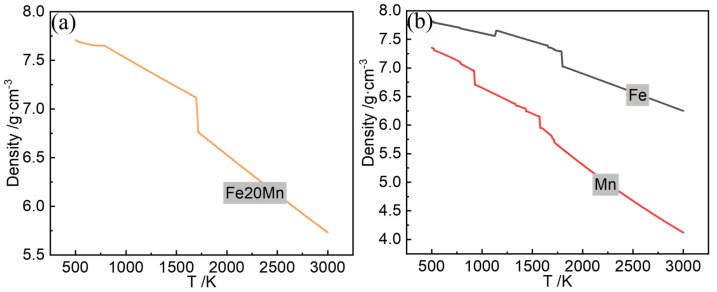
Density of different materials: (**a**) Fe20Mn pre-alloyed powder; (**b**) Fe and Mn elemental powders.

Figure 2 shows the Fe20Mn heterogeneous powder particle distribution. The Fe and Mn powders are randomly distributed on the powder bed, and the simulated heterogeneous element powder distribution proportionally generated using the DEM is in good agreement with the experimental results observed through SEM in trend, which meets the requirement of the Fe–Mn mass ratio statistically. The schematic diagram of the computational domain of SLM is shown in Figure 3. The powder data are then transferred into a CFD model of FLOW 3D to analyze the interaction between the laser beam and the powder. The laser scans the surface of the substrate along the positive direction of the *X*-axis. The Thermo-Calc v2023^®^ software is used for calculating the thermophysical properties, such as the melting and boiling temperatures of metal materials dependent on the temperatures. Table 1 lists the thermophysical properties of Fe–Mn elements and Fe20Mn pre-alloyed powders, and Figure 4 shows the change in the density of Fe20Mn pre-alloyed and Fe–Mn element materials depending on the temperature. The laser power used is 170 W, and the scanning speed is 1000 mm·s^−1^. The spot diameter is 35 μm.

## 3. Results and Discussion

### 3.1. Experiment Design and Model Verification

The high-throughput equipment developed by the China Iron and Steel Research Institute Group [24] is used to feed the heterogeneous powder bed and sample forms. The equipment consists of one IPG single-mode fiber laser with a wavelength of 1064 nm (maximum power of 500 W, minimum focused spot diameter not exceeding 0.08 mm), a control system, a shaping system, and an atmosphere protection system. The maximum printing size is 120 mm × 120 mm × 150 mm. This study considers the laser power and scanning speed as variables while keeping other parameters constant. The laser power is selected as 120, 170, and 220 W, and scanning speeds are chosen as 500, 1000, and 1500 mm·s^−1^. A total of nine sets of process parameters are designed, with sample dimensions of 9 mm × 9 mm × 8 mm. At the beginning of printing, a layer of metal powder is first pre-spread on the substrate, followed by selective melting of the metal powder controlled by a high-energy laser beam according to the information on the slicing software Magics v27. The substrate used for printing is 304 stainless steel, and the entire printing process is carried out in an argon atmosphere to prevent oxidation during the formation of the Fe–Mn alloy. Argon protective gas is injected into the printing chamber at a speed of 3 m·s^−1^ to preheat the substrate. Printing begins when the substrate temperature reaches 140 °C and the oxygen concentration in the chamber is reduced to below 0.01%.

**Figure 5:**
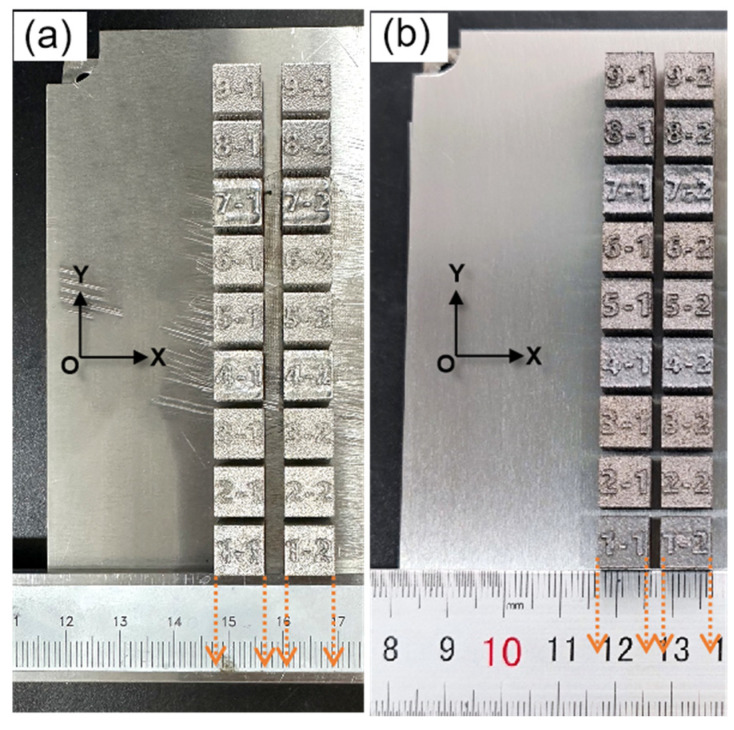
Samples of Fe20Mn prepared through the SLM process: (**a**) heterogeneous powder; (**b**) pre-alloyed powder.

The samples of Fe20Mn alloys prepared using high-throughput equipment for SLM based on elemental powder and pre-alloyed powder are shown in Figure 5a,b. The spherical Fe and Mn element heterogeneous powders and Fe20Mn pre-alloyed powder prepared through the gas atomization process are used as experimental raw materials. The distribution of each characteristic particle size measured using the Bettersize2000 laser particle size distributor (Brookfield, Shanghai, China) is shown in Table 2. The Mn powder and Fe powder based on the mass ratio (80 wt.% Fe and 20 wt.% Mn) of the elements are mixed through a mechanical mixing method. Two groups of Fe20Mn samples are prepared using the heterogeneous powder and pre-alloyed powder in the SLM process, and a total of 36 samples with a size of 9 mm × 9 mm × 8 mm are formed, as shown in Figure 5.

**Figure 6:**
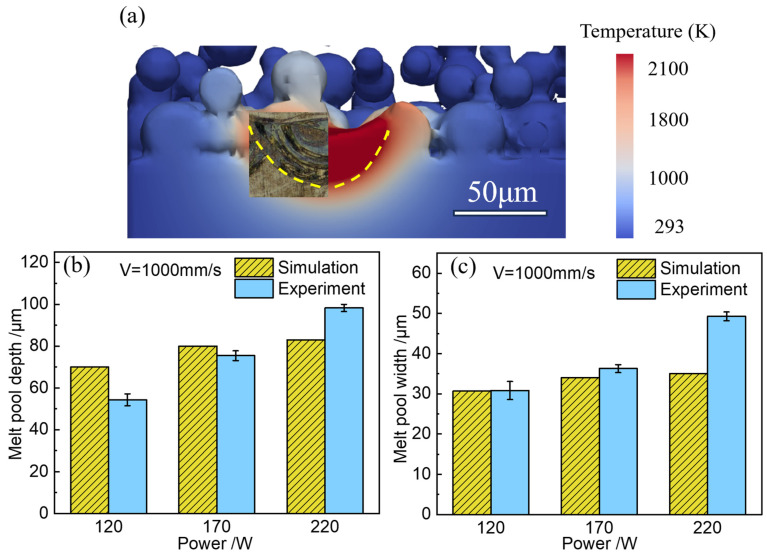
Comparison of the cross-sectional shape and size of the melt pool of the Fe20Mn heterogeneous powder: (**a**) the morphology comparison of simulation and experimental results (labeled by melting temperature of Fe20Mn); (**b**) melt pool depth; (**c**) melt pool width.

Figure 6 shows the comparison of the cross-sectional shape and the size of the melt pool from SLM experiment samples and simulated results of the Fe20Mn heterogeneous powder. For SLM-formed samples, after polishing and corrosion, the metallographic structure can be observed using a metallographic microscope to determine the morphology of the melt pool and to measure its width. It can be observed that the tendency of the predicted transient temperature contour, the maximum melting width, and the depth across varying laser powers are in good agreement with the experimental results, which partially validate the numerical model. Some deviations may be attributed to uncertain factors, such as different material surface laser absorption rates and the elements’ diffusion behaviors during the alloying for different laser powers.

### 3.2. Characteristics of Temperature and Flow Field of Heterogeneous Powder Bed

**Figure 7:**
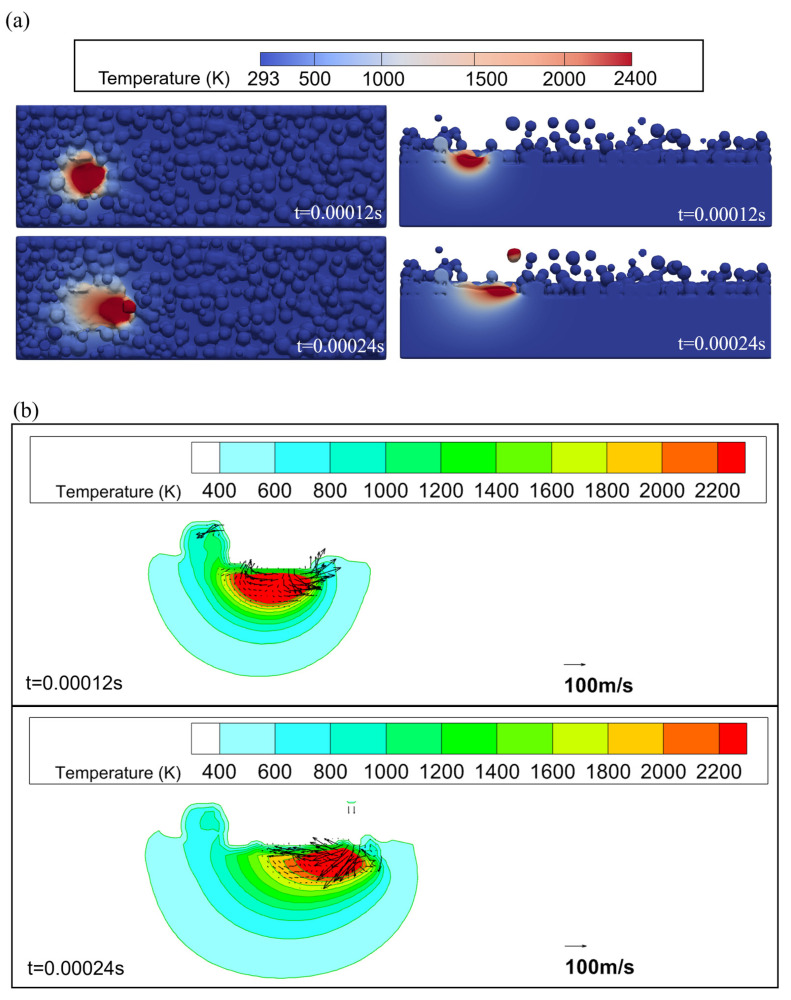
Temperature and flow fields of Fe20Mn heterogeneous powder: (**a**) temperature fields; (**b**) local enlargement of temperature and flow distribution.

Figure 7 presents the transient temperature and flow fields of the Fe20Mn heterogeneous powder at different times. Figure 7a shows the transient temperature fields of Fe20Mn heterogeneous powders’ melt pool at different times during the SLM process. The temperature field decreases gradually from the center to the surroundings. The highest temperatures are found in the zone directly exposed to the laser beam, leading to rapid melting of the substrate surface and the formation of a melt pool. From t = 0.00012 s to t = 0.00024 s, when the laser spot moves away, the surface temperature in the affected area drops rapidly. Additionally, due to the direction of laser scanning, a trailing temperature distribution in the rear and a larger temperature gradient in the front of the post-heated area are observed. Figure 7b presents the transient temperature fields and the flow fields of the Fe20Mn heterogeneous powder SLM melt pool on the X–Z cross-section. Higher velocities are observed at the melt pool’s front end at t = 0.00012 s. At t = 0.00024 s, a more intense fluid motion within the melt pool and a broader heat-affected zone for Fe20Mn heterogeneous powder can be found. Near the pool’s surface, the region directly beneath the laser withstands higher temperatures compared to the rear, driving a temperature-dependent surface-tension-driven flow pattern. This pattern transports molten metal from beneath the laser beam towards the bath’s rear, resulting in a surface profile with a suppressed front and a hump at the rear.

**Figure 8:**
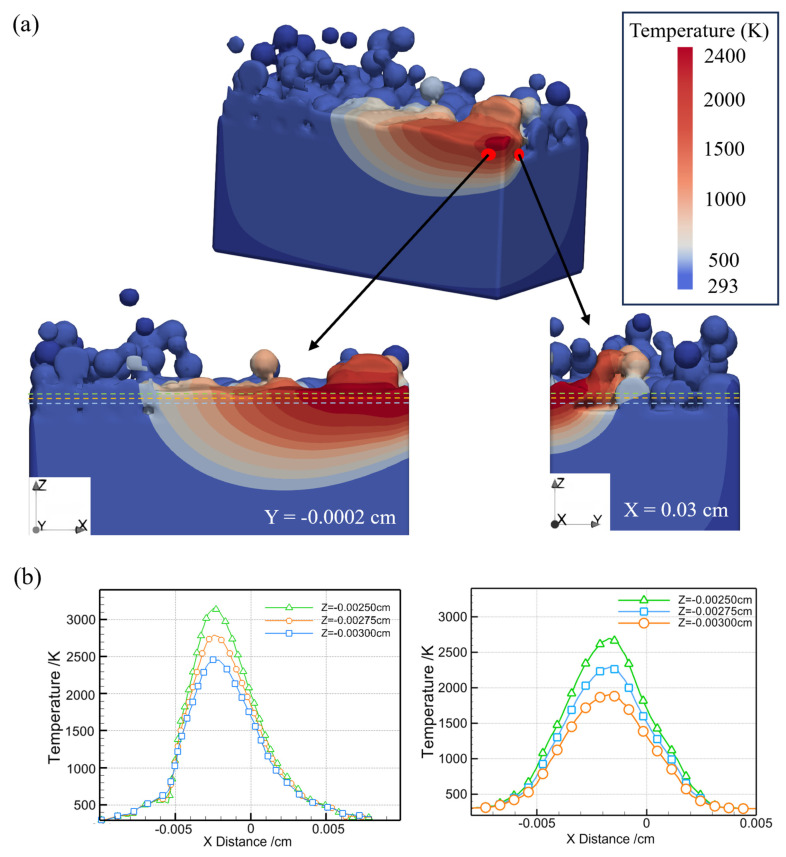
Temperature fields and the temperature profile on the cross-section of the Fe20Mn heterogeneous powder bed of the melt pool cross-section: (**a**) temperature field distribution; (**b**) temperature distribution profiles along different Z directions on the Y = − 0.0002 cm plane and X = 0.03 cm plane.

The temperature field of the Fe20Mn heterogeneous powder bed on the transverse section of the melt pool based on Y = −0.0002 cm plane and X = 0.03 cm plane is shown in Figure 8a. Figure 8b indicates the corresponding temperature profile along different Z directions. The peak temperature is near the center of the melt pool, approximately 3200 K, and there is a significant temperature difference of about 2000 K along the depth direction from the top to the bottom. Furthermore, the heat is primarily concentrated within a narrow region with a small HAZ. The simulated melt pool shape appears as a standard semi-elliptical form. In Figure 8b, along the depth direction, as one moves away from the material surface, the maximum temperature progressively decreases, indicating that the heating and cooling rates are not uniform across the different regions along the depth direction. This means that the evaporation mainly occurs on the surface of the melt pool with a high temperature.

### 3.3. Comparison and Discussion

**Figure 9:**
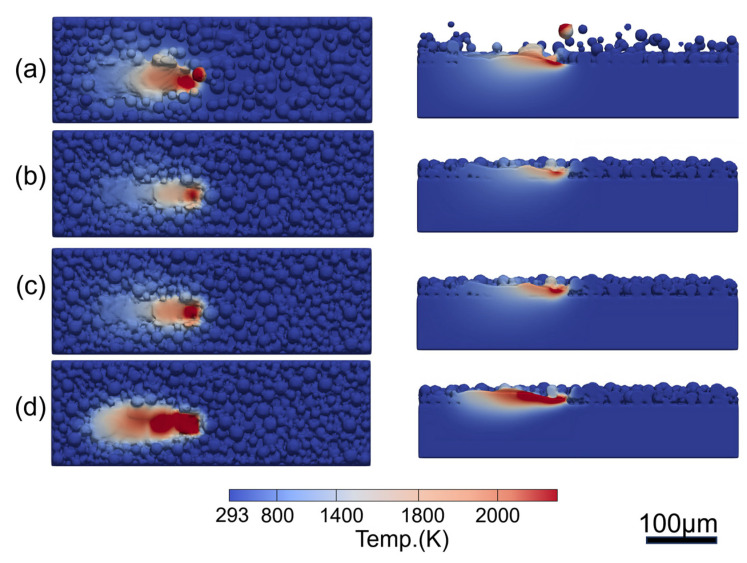
Temperature distribution for different powders at t = 0.00036 s: (**a**) Fe20Mn heterogeneous powder; (**b**) Fe20Mn pre-alloyed powder; (**c**) Fe element powder; (**d**) Mn element powder.

The predicted temperature distributions of the melt pool for the Fe20Mn heterogeneous powder, the Fe20Mn pre-alloyed powder, and the Fe and Mn element powders are shown in Figure 9. The maximum temperature for four kinds of powder materials occurs in the region near the laser beam center, which is similar to Bayat’s [20]. However, the melt zone in the SLM process for Fe20Mn heterogeneous powders is wider than that of the Fe20Mn pre-alloyed powders, resulting in slower solidification. The Mn element powder exhibits the broadest heat-affected zone during the SLM process, while the Fe element powder has the narrowest range. Different kinds of materials with various thermo-physical properties affect the size of the heat-affected zone and the volume of the melt pool during SLM processing, which may have a significant effect on the evaporation behavior in the melt pool.

**Figure 10:**
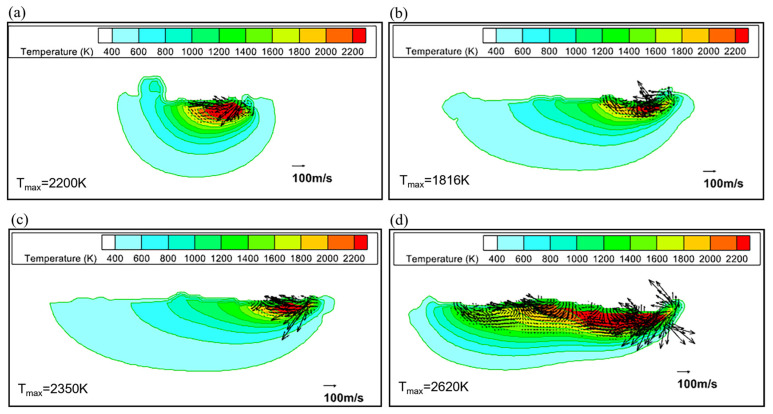
Comparison of velocity and temperature distribution in melt pool for different powder beds (Y = −0.002 cm plane): (**a**) Fe20Mn heterogeneous powder; (**b**) Fe20Mn pre-alloyed powder; (**c**) Fe element powder; (**d**) Mn element powder.

Figure 10 shows a comparison of the velocity field and temperature distribution along the longitudinal section slice in the melt pool for different powders on the Y = −0.002 cm plane. It can be seen that the Fe20Mn heterogeneous powder, Fe20Mn pre-alloyed powder, and Fe and Mn elemental powders present a complicated velocity distribution pattern in the melt pool under the same computational conditions. For the Fe20Mn heterogeneous powder, the melt pool zone with a higher temperature is large and has a different velocity distribution compared to the pre-alloyed powder. For elemental powders, the predicted velocity of the elemental Mn in the front end of the melt pool is stronger and the temperature values are higher, and the solidification is slower than that of the elemental Fe powder. The powder bed with Mn powders has a wider heat-affected zone because the Fe element has a higher thermal conductivity and melting temperature than the Mn element [25].

**Figure 11:**
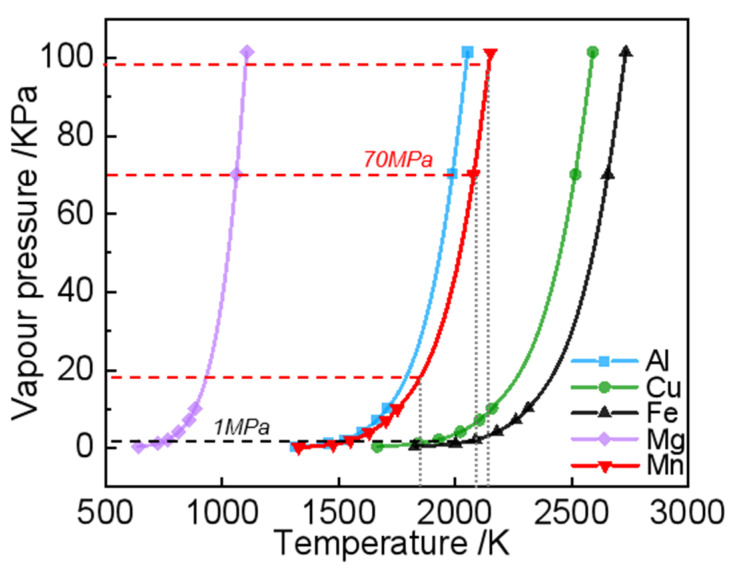
Saturated vapor pressure versus temperature for some pure metals.

Figure 11 shows the calculated saturated vapor pressure varying with temperature for various metals, according to the references [7,22,26]. At the same temperature, the vapor pressure of Mn is greater than that of Fe, and the evaporation rate of Mn is greater than that of Fe. With the increases in temperature, the saturated vapor pressure increases, which presents a greater mass loss. Lower melting points and higher saturation vapor pressures usually mean a higher evaporation tendency of the substance. In addition, the difference in the boiling points of Fe and Mn means the lower loss of Mn from the liquid Fe–Mn alloy compared to the Mn element. In the Fe–Mn alloy, the presence of Fe lowers the chemical activity of Mn, which reduces its tendency to evaporate at temperatures lower than the boiling point of pure Mn [27]. Therefore, a greater mass loss for the Mn elemental powder may be related to a wider melt pool with a high temperature and a stronger fluid flow than the Fe element, as shown in Figure 9.

**Figure 12:**
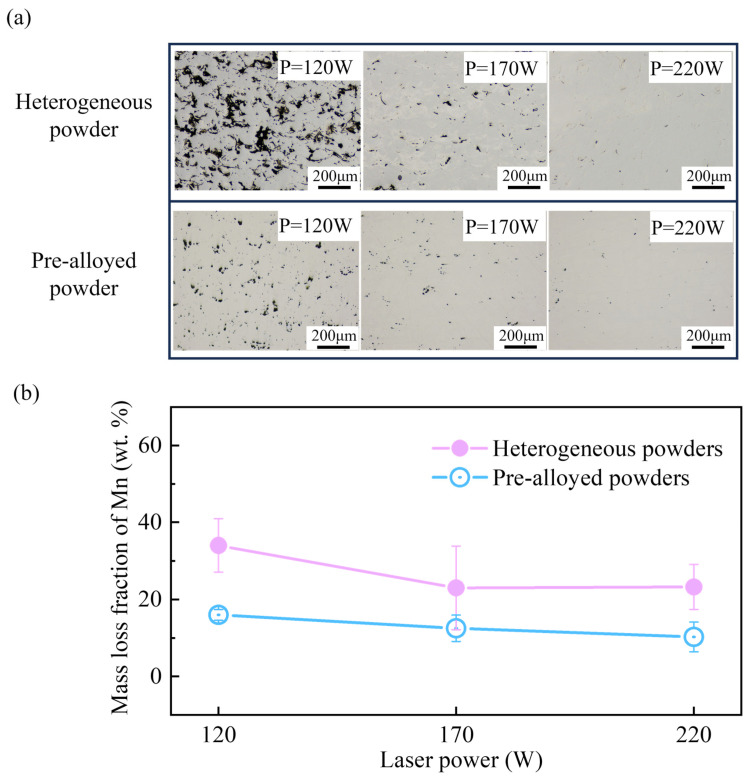
Optical microstructure and the mass loss fraction of the Mn element of Fe20Mn samples prepared using the heterogeneous and pre-alloyed powders across varying laser powers in the SLM process: (**a**) optical microstructure; (**b**) the mass loss fraction of Mn.

Moreover, it can also be seen from Figure 11 that the saturated vapor pressure is related to the material properties and the temperature. The evaporation rate of Mn is much higher than that of Fe in the mixed state of the Fe powder and the Mn powder in heterogeneous powders. Moreover, the mass loss resulting from the evaporation behavior depends on the evaporation flux ($J_{m}$), the surface area, and the duration time in the melt pool. In combination with Figure 11 and Figure 12, it can be estimated that the evaporation rate of the heterogeneous powder is higher than that of the pre-alloyed powder due to the large melt pool zone with higher temperatures.

Figure 12 shows the optical microstructure and the Mn mass loss of Fe20Mn samples experimentally prepared using the heterogeneous powder and the pre-alloyed powder at the scanning speed of 1000 mm·s^−1^ and the varying laser powers in the SLM process. The effects of Fe20Mn heterogeneous and Fe20Mn pre-alloyed powders on the optical microstructure and the elemental loss at different laser powers are obvious. With the increasing laser powers, the alloying of the heterogeneous powder and pre-alloyed powder becomes more sufficient. During the SLM process, the mass loss of the Mn element of the Fe20Mn heterogeneous powder is greater than that of the pre-alloyed powder with the change of laser powers, which can reach 17 wt.% at P = 120 W. To control the Mn content of heterogeneous powder-forming parts for multi-material additive manufacturing, more proportions of Mn should be prepared. The tendency is consistent with the numerical analysis of the effect of evaporation behavior of Fe20Mn heterogeneous powder in the SLM process. At a lower laser power (P = 120 W), the Fe20Mn heterogeneous powder does not alloy completely, and a higher mass loss of Mn may be attributed to the evaporation behavior of Mn. At P = 170 W and 220 W, it can be observed from Figure 12a that the alloying of Fe20Mn heterogeneous powder has almost finished. The change of mass loss of the Mn element with the increase in the laser power is not significant compared with P = 120 W. The present investigation helps provide a theoretical basis for laser processing parameters, the accuracy of chemical composition control, and the quality of the final product for heterogeneous powder in the SLM process.

## 4. Conclusions

In this study, a mathematics model based on computational fluid dynamics software FLOW-3D v12.0^®^ is developed to simulate the characteristics of the flow field and temperature field of Fe20Mn heterogeneous powder, Fe20Mn pre-alloyed powder, and Fe–Mn elemental powders during the SLM process. The flow field and temperature field of different kinds of powders were compared and analyzed in the SLM process. The experimental tests for the Fe20Mn heterogeneous powder and the pre-alloyed powder were performed to verify this model. Based on the analysis of the modeling results and the comparison with the experiments, the following conclusions can be drawn:

- In the Fe–Mn powder SLM process, the excessive accumulation of heat increases the maximum temperature of the melt pool, thus increasing mass loss. The heat-affected zone for the Fe20Mn heterogeneous powder with a higher temperature is large, and it has a different velocity distribution compared with the pre-alloyed powder. At P = 170 W, the predicted peak temperature of the Fe20Mn heterogeneous powder melt pool is higher than that of the pre-alloyed powder, and the difference is up to nearly 400 K.
- For Fe and Mn elemental powders in the SLM process, the Mn element powder exhibits the broadest heat-affected zone during the SLM process, while the Fe element powder has the narrowest range. The greater mass loss for Mn elemental powder may be related to a wider melt pool with a high temperature and a stronger fluid flow than the Fe element.
- For Fe20Mn heterogeneous powders, the calculated melt pool zone is large with higher temperatures, which shows a more intensive evaporation tendency compared with the Fe20Mn pre-alloyed powders. Experimentally, the mass loss of the Mn element for Fe–Mn heterogeneous powders is significantly greater than the pre-alloyed powders in the SLM process for different laser powders, up to 17 wt.% at P = 120 KW. This tendency from the experiment results is in good agreement with the numerical analysis of the effect of evaporation behavior of the Fe–Mn heterogeneous powder in the SLM process.

## Figures and Tables

**Table 1:** Thermophysical parameters of Fe, Mn, and Fe20Mn pre-alloy.

Properties	Mn	Fe	Fe20Mn Pre-Alloy
Melting temperature	1246 K	1538 K	1697 K
Boiling temperature	2061 K	2750 K	1716 K
Evaporation latent heat [9,22]	225.9 KJ/mol	340.9 KJ/mol	300.0 KJ/mol
Melting latent heat [22,23]	14.0 KJ/mol	13.8 KJ/mol	13.2 KJ/mol
Thermal conductivity	7.8 W/(m·K)	80.0 W/(m·K)	35.0 W/(m·K)

**Table 2:** Powder particle size distribution.

Material	D_10_/μm	D_50_/μm	D_90/_μm	Average Powder Size/μm
**Fe**	11.98	28.42	51.12	26.80
**Mn**	24.50	30.15	60.48	28.12
**Fe20Mn pre-alloyed powder**	20.31	37.49	61.46	39.55

## Data Availability

Data are contained within the article.
